# Arabinoxylan as well as β-glucan in barley promotes GLP-1 secretion by increasing short-chain fatty acids production

**DOI:** 10.1016/j.bbrep.2022.101343

**Published:** 2022-09-13

**Authors:** Kento Mio, Reina Ogawa, Natsuki Tadenuma, Seiichiro Aoe

**Affiliations:** a Graduate School of Studies in Human Culture, Otsuma Women's University, 12 Sanban-cho, Chiyoda-ku, Tokyo, 102-8357, Japan; b Research and Development Department, Hakubaku Co. Ltd, 4629 Nishihanawa, Chuo-shi, Yamanashi, 409-3843, Japan; c Department of Food Science, Otsuma Women's University, 12 Sanban-cho, Chiyoda-ku, Tokyo, 102-8357, Japan

**Keywords:** Barley, Dietary fiber, Arabinoxylan, β-Glucan, GLP-1, Short-chain fatty acid

## Abstract

Barley is rich in soluble dietary fiber including β-glucan and arabinoxylan. Barley β-glucan is fermented by gut bacteria and, thereby contributes to an effect on intestinal bacterial composition and short-chain fatty acids (SCFAs). It also increases GLP-1 secretion via SCFAs receptor. However, few studies have focused on barley arabinoxylan. Therefore, we have investigated the effects of arabinoxylan from barley on intestinal fermentability and GLP-1 secretion. C57BL/6J mice were fed a high-fat diet containing arabinoxylan-dominant barley flour without β-glucan (bgl) and high β-glucan-containing barley flour (BF) for 12 weeks. We conducted oral glucose tolerance test (OGTT) to measure insulin and GLP-1 concentrations. The concentration of SCFAs in the cecum contents was also determined. Furthermore, we measured mRNA expression assay GLP-1 secretion using real-time PCR. The OGTT result showed that GLP-1 concentrations at 60 min were increased in mice fed bgl and BF. Acetic acid and total SCFAs concentrations in the cecum contents were increased in both the barley groups, and butyric acid was increased in the bgl group. Furthermore, the bgl and BF groups had increased Gpr43, a receptor for SCFAs, and NeuroD which is involved in L cell differentiation. These results show arabinoxylan as well as β-glucan is involved in the SCFAs-mediated increase in GLP-1 secretion upon barley consumption.

## 1 Introduction

Barley is a cereal rich in soluble dietary fiber, the main components of which are β-glucan and arabinoxylan, both non-digestible polysaccharides. β-Glucan increases viscosity of digesta when it reaches the small intestine and delays the absorption of other nutrients [1]. As a result, postprandial blood glucose levels rise more slowly, and insulin secretion is reduced, thus improving insulin resistance. Furthermore, intake of barley β-glucan is also known to normalize blood cholesterol levels by increasing the excretion of neutral and acidic sterols, thereby suppressing visceral fat and serum cholesterol accumulation. A beneficial effect of barley consumption has been shown, especially in obese and hypercholesterolemic individuals [2,3].

Intestinal bacteria mainly ferment barley β-glucan in the cecum and colon [4], and the effects of intestinal fermentation of barley β-glucan have been studied. When β-glucan is fermented, short-chain fatty acids (SCFAs) such as acetic acid, propionic acid, and butyric acid are produced as metabolites. A meta-analysis study summarizing 14 intervention groups, including 205 participants aged 20–69 years, confirmed the effect of grain intake on SCFAs production; the results showed that cereal fiber intake increased total SCFAs (acetate, propionate, and butyrate) [5]. These SCFAs are absorbed in the intestinal tract and become a source of energy for the host. Moreover, SCFAs may also stimulate the release of the incretin hormones glucagon-like protein 1 (GLP-1) via the G protein-coupled receptors 43 (Gpr43) in L-cells of the gastrointestinal, endocrine cells. GLP-1 triggers secretion of insulin and improves insulin resistance. Therefore, these previous studies imply that intestinal fermentation of barley β-glucan suppress postprandial hyperglycemia via secretion of GLP-1.

Barley arabinoxylan is the second abundant cell wall polysaccharide after β-glucan in barley [6]. Wheat bran contains high levels of arabinoxylan, while oats contain little [7]. Although levels vary depending on the variety, barley arabinoxylan is present at 1.97–8.42% in the bran and 0.70–2.13% in the endosperm [8,9]. A previous study showed that intake of wheat-arabinoxylan modulated both gut microbiota and lipid metabolism in obese mice [10]. However, these studies used arabinoxylan derived from wheat. Few studies have shown the physiological function of arabinoxylan from barley on host metabolism. Since arabinoxylan and β-glucan have different molecular structures and viscosity, there may be differences in fermentation profile in the gastrointestinal tract and the GLP-1 secretory response. Therefore, in this study, C57BL/6J mice were fed arabinoxylan-dominant barley, which does not contain β-glucan, and barley flour, which contains high levels of β-glucan. The main purpose of this study was to investigate the effects of arabinoxylan from barley on intestinal fermentability and GLP-1 secretion.

## 2 Materials and methods

### 2.1 Sample preparation and analysis

We used high-β-glucan barley (“Beau Fiber” (BF)) and β-glucan-free barley (“Shikoku-hadaka 84” (bgl)). The nutritional composition of each barley flour is shown in Supplemental Table 1. The concentration of β-glucan in barley flour was determined by the AOAC 995.16 methods [11]. Total dietary fiber was determined by AOAC 991.43 methods [12]. Proteins were analyzed using Kjeldahl method [13]. Lipid were extracted by acid hydrolysis with hydrochloric acid. Extracted lipids were dried using a rotary evaporator, and their yield was calculated. Soluble dietary fiber fractions (SDF) were extracted from BF and bgl flour and analyzed for saccharide composition (Supplemental Fig. 1). 100 ml of hexane was added to 10 g of barley flour, and the mixture was stirred for 8 h. The residue was then collected by centrifugation, and hexane was removed. This defatted barley flour was used to extract the SDF following a previous study [14]. Ten times the volume of distilled water was added to the residue and adjusted to pH 10 with Na_2_CO_3_. The solution was then centrifuged, and the resulting supernatant was adjusted to pH 4.5 with 2 M HCl and centrifuged again. Two times the volume of ethanol was added to the supernatant with stirring, and the solution was left overnight to precipitate the gum material. The next day, the solution was centrifuged and resulting residue was washed with ethanol on glass filter to obtain barley SDF. SDF was acid-degraded with sulfuric acid to extract monosaccharides, and the neutralized solution was analyzed using GC/MS.

### 2.2 Animals and research design

The animal protocol was approved by the Animal Research Committee of Otsuma Women's University (Tokyo, Japan), and implemented with regulations (No. 21003). We used four-week old C57BL/6J male mice purchased from Charles River Laboratories Japan, Inc. (Yokohama, Japan). Each mouse was acclimatized for 7 days on standard chow diet (NMF, Oriental Yeast Co., Ltd., Shiga, Japan) in plastic cages on a 12-h light/dark cycle (lights on at 7:30) at a temperature of 22 ± 1 °C and humidity of 50 ± 5%. After acclimatization, mice were randomly divided into three groups according to their body weight. Mice were individually placed and then fed the experimental diet and water ad libitum for 12 weeks. The composition of the experimental diet is shown in Table 1. The experimental diets were 50% fat energy and prepared by the addition of lard to the AIN-93G diet. The control diets (C) were supplemented with insoluble fiber cellulose to give a total dietary fiber content of 5%. β-Glucan-free barley diets (bgl) and high-β-glucan barley diets (BF) were supplemented with bgl and BF to give the same fiber content as the C group. During the experimental study, the food intake and body weight of mice were monitored two or three times a week. In the 11th week of the study period, OGTT was performed. Each mouse was fasted for 8 h, and then orally given 20% glucose solution as 1.5 g/kg body weight. Blood samples were collected from the tail at 0 (fasted), 15, 30, 60, and 120 min after administration of glucose solution and blood glucose levels determined using enzymatic methods (Glutest Ace R, Sanwa Kagaku Kenkyusyo Co., Ltd). The concentrations of total GLP-1 and insulin were determined at 0, 15, 30, and 60 min after administration of glucose solution using Enzyme-Linked Immunosorbent Assay (ELISA), namely the Mouse Insulin ELISA Kit (Shibayagi Co., Ltd.) and the GLP-1 ELISA kit (FUJIFILM Wako Pure Chemical Corporation), respectively. The area under the blood concentration-time curve (AUC) was calculated using the values of blood glucose, insulin, and total GLP-1 obtained for each period. On the last day of the examination, mice were sacrificed by isoflurane/CO_2_ anesthesia after fasting for 8 h. The weights of liver, cecal contents, and adipose tissue were measured. Cecal contents were stored at −30 °C until SCFA analysis. Ileum were stored in RNA later RNA Stabilization Reagent (Qiagen) at −30 °C until real time-PCR analysis.

**Table 1:** Compositions of the experimental Control and barley diets.

(g/kg diet)			
	Control diet	bgl diet	BF diet
Casein	200.0	175.1	171.7
Corn starch	132.0	107.3	132.0
Dextrinized corn starch	197.5	0.0	13.9
Sucrose	100.0	100.0	100.0
Soybean oil	70.0	70.0	70.0
Lard	200.0	194.0	190.9
Cellulose	50.0	31.8	–
Beau Fiber (BF)	–	–	271.2
Shikoku hadaka S84 (bgl)	–	271.2	–
AIN-93G mineral mixture	35.0	35.0	35.0
AIN-93 vitamin mixture	10.0	10.0	10.0
l-Cystine	3.0	3.0	3.0
Choline bitartrate	2.5	2.5	2.5
*t-B*utylhydroquinone	0.014	0.014	0.014

### 2.3 Short-chain fatty acids (SCFAs) in cecum contents

The concentration of SCFAs and organic acids in cecal contents of the mice were determined using GC/MS (GC/MS, 7890B GC system equipped with a 5977A MSD; Agilent) according to a previous study [15]. The concentrations of SCFAs were calculated by comparing their peak areas with the internal standard (100 μM crotonic acid).

### 2.4 Real-time PCR about L cell function

Total RNA in the ileum was extracted using a RNeasy Mini Kit (Qiagen), and cDNA synthesized from RNA. Expression of mRNA relating to L cell function was determined by real-time PCR using an Applied Biosystems Quant3 Real-Time PCR System and PowerUp SYBR Green Master Mix (Thermo Fisher Scientific). RNA primer sequences are shown in Supplemental Table 2. The 2^–ΔΔCT^ method was used for relative mRNA expression analysis. We used 36B4 as a reference gene and calculated ΔCT compared to 36B4. Next, we calculated ΔΔCT as the difference between ΔCT for the C group and other groups (bgl, BF) in terms of cDNA solution added to each primer. Relative expression levels are presented as fold changes relative to the C group (arbitrary units).

### 2.5 Counts of gut microbiota in cecal digesta

The counts of major gut microbiota in the cecum were analyzed by real-time PCR according to a previous study [16]. Total DNA of the cecum digesta was extracted using QIAamp® Fast DNA Stool Mini kit (Qiagen). DNA was mixed with PowerUp SYBR Green Master Mix and bacterial primers at the genus level (Supplemental Table 3). This mixture solution was amplified, and threshold cycle (Ct) values obtained. Similarly, we prepared standard DNA solution by serially diluting each standard bacterial strain, amplified and obtained Ct values (Supplemental Table 3). A calibration curve was prepared from the Ct values of the standard strains, and colony-forming units (CFU) in the cecum digesta calculated.

### 2.6 Statistical analysis

All statistical analysis was performed using R studio software (ver. 1.3.1093; R-Tools Technology Inc., Richmond Hill, ON, Canada). Sample sizes were calculated from our previous study mice data which was based on differences in cecum total SCFAs concentrations of 3.2 μmol/g with a standard deviation (SD) of 2.0 μmol/g [17]. The sample size was then calculated with the result that 7 mice were required in total (type I error (α) = 0.05, 1 − β = 0.80); a total of 24 mice were used, n = 8 per group. All data are presented as mean ± standard error of the mean (SE). Significant differences between the experimental groups were determined by Dunnett's multiple comparison test with the control group. A p value < 0.05 is considered statistically significant.

## 3 Results

### 3.1 Compositional analysis of water-soluble fiber fractions extracted from barley flour

**Fig. 1:**
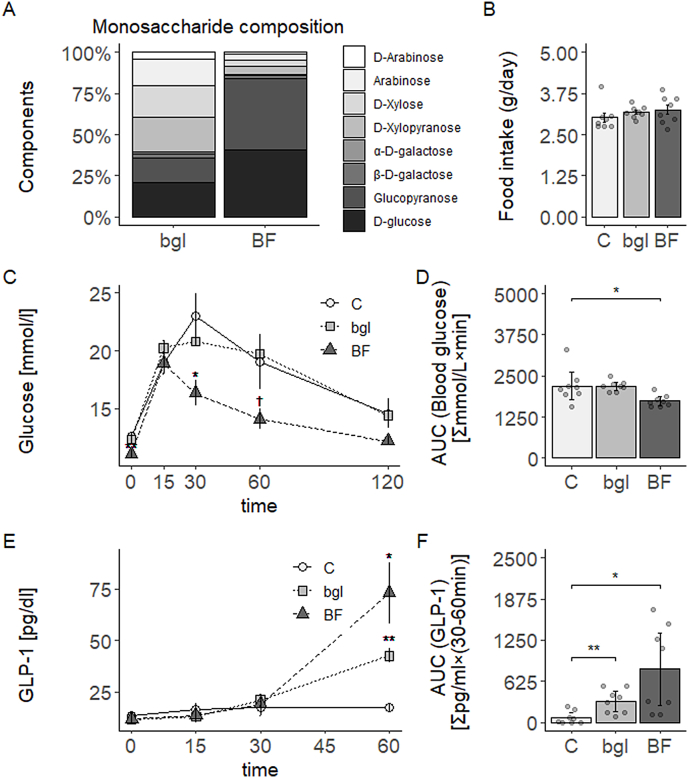
Soluble fiber composition of barley flours, feed intake of mice fed experimental diet, and blood glucose and GLP-1 responses during OGTT (A) Compositional analysis of water-soluble fiber fractions extracted from bgl and BF flours. (B) Feed intake of mice fed a high-fat diet containing bgl and BF for 12 weeks. (C–D) Glycemic response and AUC during OGTT performed at week 11 of the study. (E–F) Concentration of GLP-1 in the OGTT performed at week 11 of the study and AUC calculated from 30 min to 60 min. Results are shown as the mean ± SE (n = 8 mice per groups). *p < 0.05, **p < 0.01, †p = 0.06; assessed with Dunnett's multiple comparison test with the C group.

Soluble fiber components of different varieties of barley were analyzed using GC/MS (Fig. 1A, Supplemental Table 4). Glucose, which is the β-glucan fraction, accounted for 84.4% of the total SDF in BF; in bgl was 35.7%. Because bgl flour does not contain β-glucan (Supplemental Table 1), the glucose fractions in bgl may be derived from another soluble fiber. Arabinose and xylose, which are the arabinoxylan fractions, accounted for 4.9% and 8.3% of BF; bgl were 20.4% and 40.4%, respectively. A previous study reported arabinose and xylose ratios of 26.3% and 38.3%, respectively, for whole-grain barley extracted with alkali [18], results which are mostly consistent with this study. Therefore, SDF of BF had a high purity of β-glucan, and bgl had high arabinoxylan.

### 3.2 Arabinoxylan contributes to the increase in GLP-1 secretion upon barley flour consumption

C57BL/6J mice were fed a high-fat diet with bgl and BF flour for 12 weeks. There was no difference in food intake of the mice that consumed each test diet with them consumed equivalent amounts of energy (Fig. 1B). Final weight, body weight gain, food efficiency ratio, and abdominal fat were significantly lower in the BF group compared to the C group (p < 0.05) (Supplemental Table 5). In the OGTT, blood glucose levels during fasting, after glucose solution administration at 30 min and AUC were significantly lower in the BF group compared to the C group (p < 0.05) and tended to the lower at 60 min (p = 0.06) (Fig. 1C-D). Insulin levels during fasting were significantly lower in the BF group compared to the C group (p < 0.05) and tended to the lower at 15 min (p = 0.06) (Supplemental Fig. 2A). There was no significant difference in GLP-1 concentration at fasting and 15 and 30 min after glucose solution administration. However, GLP-1 concentrations at 60 min were significantly higher in the BF and bgl groups than in the C group (Fig. 1E). The AUC from 30 to 60 min after glucose administration was significantly higher in both the bgl and BF groups than in the C group (Fig. 1F). Therefore, the increase in GLP-1 secretion caused by barley consumption was a function not only of β-glucan but also of arabinoxylan.

### 3.3 Barley flour intakes with different fiber sources increase short-chain fatty acids

**Fig. 2:**
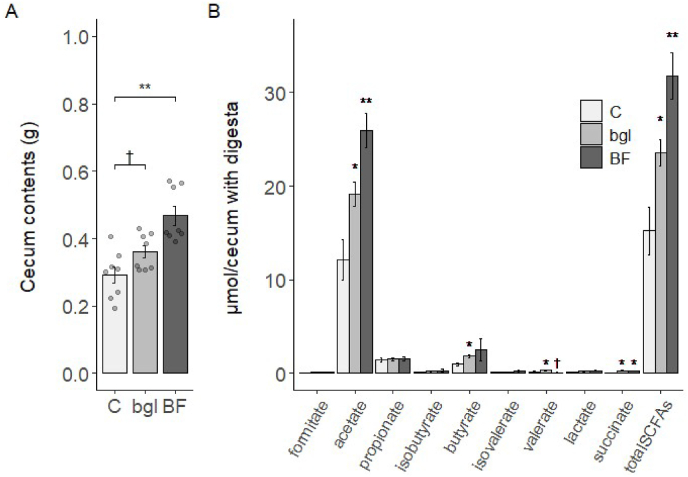
Cecum weight and concentration of SCFAs in cecum contents of mice (A) Cecum weight of mice with cecum contents. (B) Concentration of SCFAs in cecum contents of mice. Results are shown as the mean ± SE (n = 8 mice per groups). *p < 0.05, **p < 0.01, †p = 0.06; assessed with Dunnett's multiple comparison test with the C group.

We investigated the effects of barley flour consumption on intestinal fermentation. The weight of the cecum with contents significantly increased in the BF group compared to the C group (p < 0.05), and the bgl group also tended to increase (p = 0.10) (Fig. 2A). The concentrations of acetate, succinate, and total SCFAs in the cecum content were significantly higher in the bgl and BF groups compared to the C group (p < 0.05) (Fig. 2B). The concentrations of butyrate and valerate were significantly higher in the bgl group compared to the C group (p < 0.05). Thus, both arabinoxylan and β-glucan in barley result in increased metabolites in the gut. Furthermore, the observed increased in intestinal bacteria differ with the different sources of soluble fiber. The counts of *Clostridum leptum* group were significantly higher in the BF group compared to the C group (p < 0.05). The counts of *Lactobacillus* were significantly higher in the bgl group compared to the C group (p < 0.05) (Supplemental Table 6).

### 3.4 Effect of each barley flour on the expression levels of genes involved in GLP-1 secretion and L-cell differentiation

**Fig. 3:**
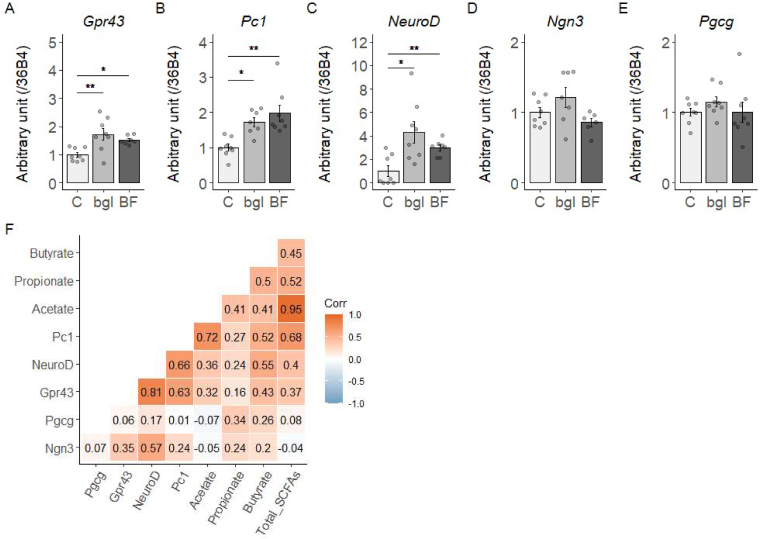
mRNA expression levels related to L-cell function in the ileum (A) mRNA expression levels of genes related to L cells in the ileum. (B) Correlation analysis between mRNA expression levels for L cells and SCFAs. Numbers are correlation coefficients between each factor. Results are shown as the mean ± SE (n = 8 mice per groups). *p < 0.05, **p < 0.01; assessed with Dunnett's multiple comparison test with the C group.

We investigated the level of mRNA for genes related to L cell function and GLP-1 secretion in mice. Expression of Gpr43, a type of G protein-coupled receptor, were significantly increased in the bgl and BF groups compared to the C group (p < 0.05) (Fig. 3A). Expression of Pc1, a rate-limiting enzyme that converts proglucagon (Pgcg) to GLP-1, was significantly increased in the bgl and BF groups compared to the C group (Fig. 3B). Expression of NeuroD, which is involved in L cell differentiation was also significantly increased in the bgl and BF groups compared to the C group (p < 0.05) (Fig. 3C). There was no significant difference in the expression level of Ngn3 and Pgcg in each group (Fig. 3D-E). Moreover, Pc1 was positively correlated with acetate, butyrate, and total SCFAs (p < 0.05). Similarly, NeuroD and Gpr43 were positively correlated with butyrate (p < 0.05) (Fig. 3F). These results indicate that arabinoxylan can affect L-cell differentiation and GLP-1 secretion genes via an increase in SCFAs, even if barley does not contain β-glucan.

## 4 Discussion

In this study, we investigated the effects of barley arabinoxylan on intestinal fermentability and GLP-1 secretion in mice fed β-glucan-free barley. The results show that barley arabinoxylan intake increases SCFAs in the intestine and enhances GLP-1 secretion, as does barley β-glucan. Moreover, we show that barley arabinoxylan can affect L-cell differentiation and GLP-1 secretion genes via increased SCFAs. To our knowledge, the present study is the first to show an increase in GLP-1 secretion via increased SCFAs upon intake of arabinoxylan derived from barley.

In the OGTT, GLP-1 levels were increased in the barley groups after glucose administration. barley-fed to GLP-1 concentrations increased dramatically in both barley-fed groups 60 min after glucose administration, suggesting GLP-1 was efficiently secreted in response to elevated blood glucose levels. A previous study indicated that C57BL/6J mice fed barley flour (2% β-glucan) with a high-fat diet increased GLP-1 secretion and improved insulin sensitivity [19]. Thus, barley flour high in β-glucan may improve glucose tolerance through increased GLP-1 secretion. However, the effect was also observed for barley flour which does not contain β-glucan (bgl group). A previous study reported that overweight women (BMI 25.0–29.9 kg/m^2^) increased postprandial GLP-1 by intake of a cereal containing arabinoxylan from wheat compared to a low fiber cereal [20]. In contrast, another study showed that pigs fed diets containing 5% arabinoxylan (from wheat) and 5% β-glucan (from oats) did not change GLP-1 concentrations in OGTT [21]. These previous studies have used wheat-derived arabinoxylan in their experiments. The present study is the first evidence that barley-derived arabinoxylan intake increases GLP-1 secretion in the OGTT. However, 35.7% of the SDF extract of bgl likely due to glucose derived from multiple polymers, except for β-glucan (Supplemental Table S4). Therefore, these polymers may also have affected GLP-1 secretion. Moreover, in the bgl group, the suppression of postprandial blood glucose rise could not be confirmed. The discrepancy may be due to the lower soluble fiber content in the bgl group compared to the BF group. Because equal amounts of each barley flour were supplemented with the experimental diets, the amount of soluble dietary fiber differed. Moreover, GLP-1 promotes insulin secretion in a glucose-dependent manner [22], but insulin concentrations at 60 min of sugar administration did not increase in any group. Further studies are needed to determine the effects of arabinoxylan and β-glucan on insulin secretion.

The present study shows that barley-derived arabinoxylan as well as β-glucan contributes to an increase in SCFAs. This can be attributed to the fermentation properties of xylose and arabinose. A previous study indicated that xylose increased butyrate in the ileum while arabinose caused an increase in the colon [23]. Moreover, another study which used a cumulative gas generation method using pig feces to examine the kinetics of fermentation and end products after 48 h [24], showed that the ratio of SCFAs product was similar for arabinoxylan and β-glucan substrates. This study suggests that the increase in SCFAs upon barley consumption is not necessarily dependent solely on β-glucan. Furthermore, the expression of the SCFAs receptor, Gpr43, was significantly increased in both barley groups compared to C group. Activation of Gpr43 is involved in SCFAs-stimulated GLP-1 secretion [25]. The positive correlation found between Gpr43 and butyrate and acetate data (Fig. 3F) supports this hypothesis. Moreover, the mRNA expression level of NeuroD was also significantly increased in the bgl and BF groups. Increased GLP-1 secretion in the OGTT may be due to increased L-cell counts in the ileum. In our previous study data indicated that high β-glucan barley consumption increased ileal NeuroD and cecal GLP-1 pool size [26]. The current study shows similar results, suggesting that barley-derived arabinoxylan may also increase L-cell numbers by increasing SCFAs.

The microbiota in the cecal contents which increased in the BF group was different to the C group showing increased *Clostridium leptum* group. The *Clostridium leptum* group ferment indigestible carbohydrates to produce SCFAs [27]. In contrast, the bgl group showed increased *Lactobacillus*. *Lactobacillus* are resident anaerobic lactic acid bacteria that heteroferment sugars and produce lactic acid and acetic acid [28]. A previous *in vitro* study reported that arabinoxylan extract from wheat supported the growth of *Lactobacillus* and *Bifidobacterium* and the growth rate was higher than with inulin [29]. Another *in vivo* study showed that rats fed high-fat diets supplemented with arabinoxylan showed increased *Lactobacillus* in the colon [30]. The present study is consistent with these previous studies by showing that arabinoxylan from barley also selectively increased *Lactobacillus* spp. In this study, different bacteria selectively fermented β-glucan and arabinoxylan. This may be due to the difference in the fermentation rate or fermenting substrates of arabinoxylan and β-glucan. The structure of fermentable substrates, such as polymerization degree and molecular weight, affects the fermentation rate [31]. Because *Lactobacillus* is an indigenous anaerobic *Lactobacillus*, arabinoxylan may be mainly fermented in the proximal colon. In contrast, because *Clostridium leptum* group is anaerobic, it is suggested that β-glucan may be fermented in the distal colon. Future research is needed to clarify the fermentation characteristics of arabinoxylan derived from barley flour. Moreover, previous *in vivo* study indicated that in rats-fed barley flour rich in soluble fibers such as β-glucan, resistant starch, and fructan caused increases in the fermentation capacity of the entire digestive tract more than other barley flours [4]. Therefore, barley flour rich in β-glucan and arabinoxylan may increase the diversity of gut microflora. In conclusion, we demonstrate that two fermentable dietary fibers in barley affect physiological function through intestinal fermentation.

## Funding

This work was supported by the funding from Hakubaku Co., Ltd.

## Declaration of competing interest

The authors declare the following financial interests/personal relationships which may be considered as potential competing interests: Seiichiro Aoe reports financial support was provided by Hakubaku Co., Ltd. Kento Mio reports a relationship with Hakubaku Co., Ltd that includes: employment.

## Data Availability

No data was used for the research described in the article.
