# Supplementary material for: Arabinoxylan as well as β-glucan in barley promotes GLP-1 secretion by increasing short-chain fatty acids production
Source: Biochem Biophys Rep. 2022 Sep 13;32:101343. doi: 10.1016/j.bbrep.2022.101343 (PMC9482107; doi:10.1016/j.bbrep.2022.101343)
Supplement: Multimedia component 1 [file mmc1.docx]

Supplemental table S1: The nutrient components of barley flour.

| (g/100g dw) | bgl | BF |
| --- | --- | --- |
| Moisture | 11.1 | 8.8 |
| Ash | 1.0 | 1.0 |
| Fat | 3.4 | 2.2 |
| Protein | 8.4 | 9.4 |
| Ash | 1.0 | 1.2 |
| Total starch | 68.4 | 59.0 |
| Total dietary fiber | 6.7 | 18.4 |
| β-(1-3)-(1,4) Glucan | 0.0 | 8.0 |

Supplemental table S2: Primer sequence for real-time PCR.

| gene symbol | Forward | Reverse |
| --- | --- | --- |
| *Gpr43* | 5'-GGGATCTGGGTCACATGCTTAT-3 | 5'-ATGTCAGACAGACGGGTACCAA-3' |
| *Pc1/3* | 5'-AGACAGCATTACACCATCTCTA-3' | 5'-AGAACACTTCTCTGCATACCAAGGT-3' |
| *Pgcg* | 5'-ATTGCCAAACGTCATGATGA-3' | 5'-GGCGACTTCTTCTGGGAAGT-3' |
| *Neuro3* | 5'-CTTGGCCAAGAACTACATCTGG-3' | 5'-CGTGTTTGAAAGAGAAGTTGCC-3' |
| *Ngn3* | 5'-AAGAGCGAGTTGGCACTCAGC-3' | 5'-AAGCTGTGGTCCGCTATGCG -3' |
| *36B4* | 5'-GGCCCTGCACTCTCGCTTTC-3 | 5'-TGCCAGGACGCGCTTGT-3' |

Supplemental table S3: List of standard bacterial strain and primer sequence for real time PCR.

| Genus | Fw/Rv | Primer sequence | Standard bacteria | Strain No. | Reference |
| --- | --- | --- | --- | --- | --- |
| *Bacteroides* | Fw | 5'-GTCAGTTGTGAAAGTTTGC-3' | *Bacteroides fragilis* | JCM11019 | Bernard AE et al: Appl Environ Microbiol, 66: 1587-94, 2000. |
|  | Rv | 5'-CAATCGGAGTTCTTCGTG-3' |  |  |  |
| *Lactobacillus* | Fw | 5'-TGGAAACAGGTGCTAATAGGC-3' | *Lactobacillus ramnosus* | ATCC8530 | Roy Byun et al: J Clin Microbiol, 42(7): 312836, 2004. |
|  | Rv | 5'-GTCCATTGTGGAAGATTCCC-3' |  |  |  |
| *Bifidobacterium* | Fw | 5'-AGGGTTCGATTCTGGCACAG-3' | *Bifidobacterium longum* | JCM1217 | Kok RG et al: Appl Environ Microbiol, 62: 3668-72, 1996. |
|  | Rv | 5'-CATCCGGCATTACCACCC-3' |  |  |  |
| *Blautia_cocoides* | Fw | 5'-AAATGACGGTACCTGACTAA-3' | *Clostridium coccoides* | JCM1395T | Matsuki et al: Appl Environ Microbiol, 70: 7220-8, 2004. |
|  | Rv | 5'-CTTTGAGTTTCATTCTTGCGAA-3 |  |  |  |
| *Clostridium_leptum* | Fw | 5'-GCACAAGCAGTGGAGT-3' | *Ruminococcus albus* | JCM14654 | Matsuki et al: Appl Environ Microbiol, 70: 7220-8, 2004. |
|  | Rv | 5'-CTTCCTCCGTTTTGTCAA-3' |  |  |  |
| *Prevotella* | Fw | 5'-CACCAAGGCGACGATGA-3' | *Prevotella malaninogenica* | JCM6325 | Larsen et al: PLos ONE, 5: e9085, 2010. |
|  | Rv | 5'-GGATAACGCCTGGACCT-3' |  |  |  |

Supplemental table S4: Compositional analysis of water-soluble fiber fractions extracted from bgl and BF flours.

| Component (%) | bgl | BF |
| --- | --- | --- |
| Total arabinose | 20.4 | 4.9 |
| (D-arabinose) | (4.3) | (1.0) |
| (Arabinose) | (16.1) | (3.8) |
| Total xylose | 40.4 | 8.3 |
| (D-xylose) | (18.8) | (3.7) |
| (D-xylopyranose) | (21.6) | (4.6) |
| Total galactose | 1.7 | 2.5 |
| (α-D-galactose) | (0.9) | (1.0) |
| (β-D-galactose) | (2.7) | (1.5) |
| Total glucose | 35.7 | 84.4 |
| (Glucopyranose) | (14.9) | (43.9) |
| (D-glucose) | (20.8) | (40.5) |

Supplemental table S5: Body weight, feed intake, and organ weights in mice fed experimental diets.

|  | C | bgl | BF | *p* value  C – bgl | *p* value  C – BF |
| --- | --- | --- | --- | --- | --- |
| Initial weight (g) | 19.2 ± 0.37 | 19.2 ± 0.30 | 19.2 ± 0.28 | 0.999 | 0.999 |
| Final weight (g) | 42.7 ± 1.38 | 41.6 ± 0.79 | 38.3 ± 1.03 | 0.686 | 0.016 |
| Body weight gain (g/d) | 0.28 ± 0.02 | 0.26 ± 0.01 | 0.23 ± 0.01 | 0.440 | 0.018 |
| Feed intake (g/d) | 3.02 ± 0.14 | 3.19 ± 0.06 | 3.26 ± 0.15 | 0.533 | 0.315 |
| Feed efficiency ratio (%) | 9.63 ± 0.45 | 8.42 ± 0.36 | 7.35 ± 0.68 | 0.190 | 0.001 |
| Liver weight (g) | 1.53 ± 0.09 | 1.60 ± 0.12 | 1.29 ± 0.04 | 0.784 | 0.143 |
| Cecum with contents (g) | 0.29 ± 0.02 | 0.36 ± 0.02 | 0.47 ± 0.03 | 0.100 | <.001 |
| Retroperitoneal fat (g) | 0.98 ± 0.06 | 0.95 ± 0.04 | 0.79 ± 0.04 | 0.820 | 0.020 |
| Epididymal fat (g) | 2.52 ± 0.05 | 2.35 ± 0.08 | 2.21 ± 0.13 | 0.332 | 0.050 |
| Mesenteric fat (g) | 1.06 ± 0.14 | 1.11 ± 0.10 | 0.70 ± 0.07 | 0.907 | 0.049 |

Data are presented as mean ± SE (n=8).

Feed efficiency ratio = body weight gain / food intake × 100

Abbreviations: C, control group; bgl, bgl flour group; BF, BF flour group.

Supplemental table S6: Counts of the bacterial groups in the cecum among the experimental groups.

| Log/CFU g | C | bgl | BF | *p* value  C – bgl | *p* value  C – BF |
| --- | --- | --- | --- | --- | --- |
| *Bacteroides* | 9.50 ± 0.19 | 9.11 ± 0.15 | 9.91 ± 0.15 | 0.213 | 0.187 |
| *Bifidobacterium* | 8.56 ± 0.19 | 8.72 ± 0.12 | 8.10 ± 0.34 | 0.727 | 0.438 |
| *Lactobacillus* | 9.01 ± 0.24 | 10.30 ± 0.22 * | 8.71 ± 0.34 | 0.004 | 0.701 |
| *Blautia_cocoides* | 8.90 ± 0.15 | 9.10 ± 0.15 | 8.79 ± 0.14 | 0.550 | 0.806 |
| *Clostridium_leptum* | 10.09 ± 0.15 | 10.24 ± 0.12 | 10.97 ± 0.11 * | 0.659 | 0.001 |
| *Prevotella* | 5.74 ± 0.05 | 5.69 ± 0.06 | 5.84 ± 0.09 | 0.786 | 0.581 |

Data are presented as mean ± SE (n=8).

Abbreviations: C, control group; bgl, bgl flour group; BF, BF flour group.

** p<0.01; assessed with Dunnett's multiple comparison test with the C group.

Supplemental figure S1: Flow chart of extraction of soluble fiber fraction from barley flour and isolation of monosaccharide


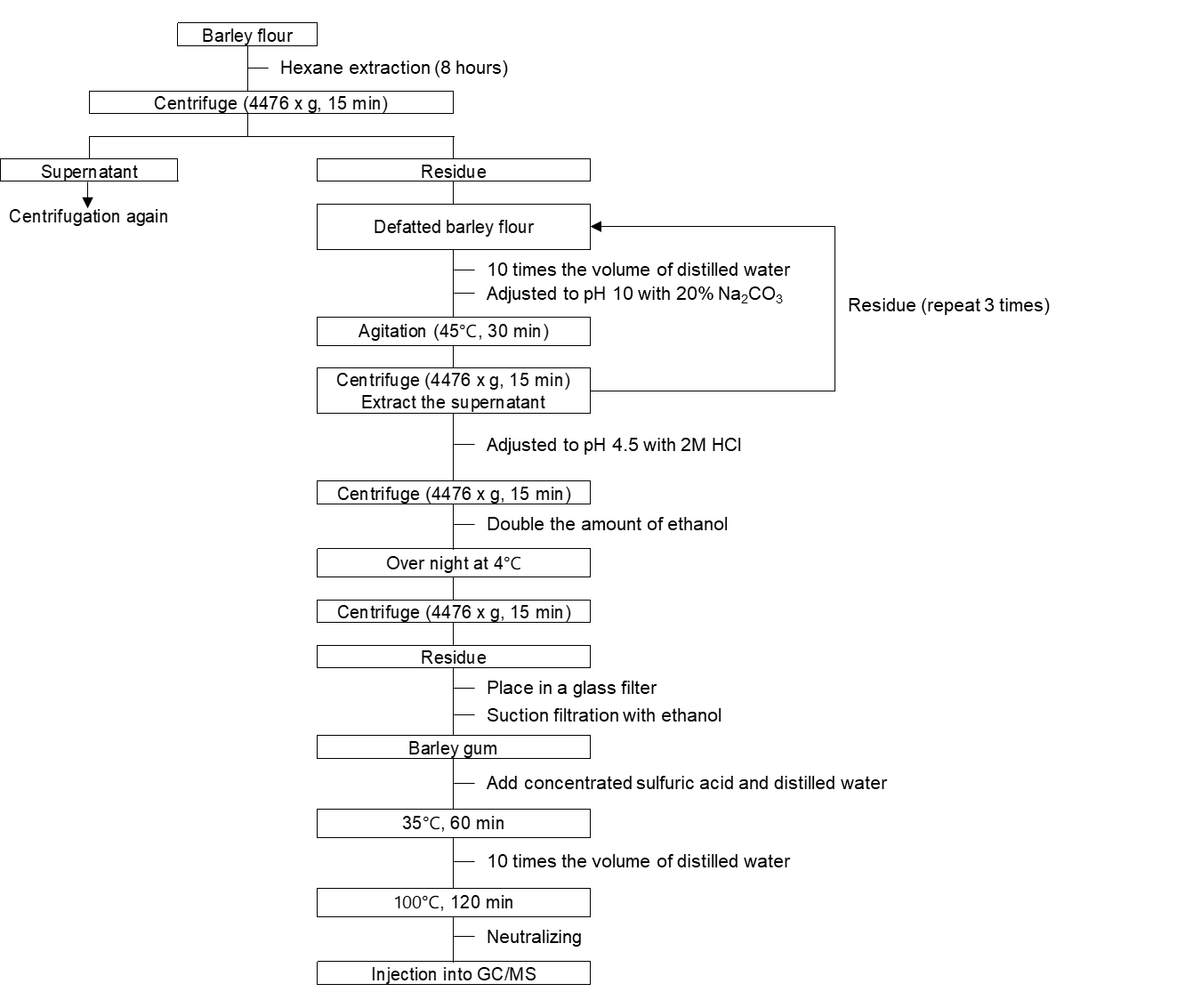


Supplemental figure S2: Concentration of insulin in the OGTT performed at week 11 of the study and AUC.


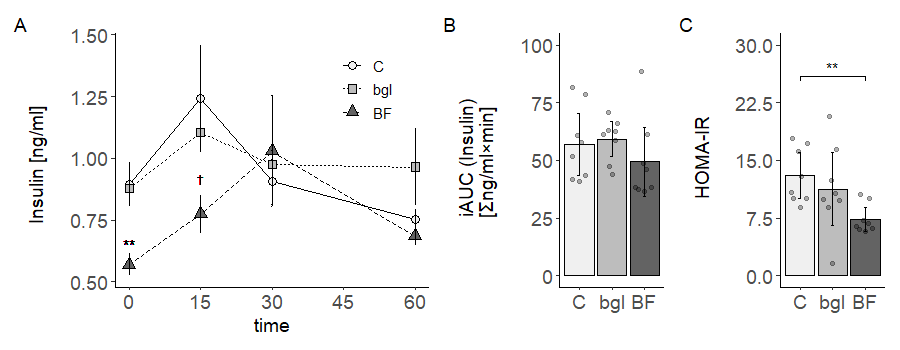


Results are shown as the mean ± SE (n=8 mice per groups).

*p < 0.05, **p<0.01; assessed with Dunnett's multiple comparison test with the C group.

Abbreviations: C, control group; bgl, bgl flour group; BF, BF flour group.
